# PROState Pathway Embedded Comparative Trial: The IP3-PROSPECT study

**DOI:** 10.1016/j.cct.2021.106485

**Published:** 2021-08

**Authors:** E.J. Bass, N. Klimowska-Nassar, T. Sasikaran, E. Day, F. Fiorentino, M.R. Sydes, M. Winkler, N. Arumainayagam, B. Khoubehi, A. Pope, H. Sokhi, T. Dudderidge, H.U. Ahmed

**Affiliations:** a Imperial Prostate, Division of Surgery, Department of Surgery and Cancer, Faculty of Medicine, Imperial College London, London, UK; b Imperial Urology, Division of Cancer, Cardiovascular Medicine and Surgery, Imperial College Healthcare NHS Trust, London, UK; c Department of Surgery and Cancer, Faculty of Medicine, Imperial College London, London, UK; d Imperial Clinical Trials Unit, School of Public Health, Faculty of Medicine, Imperial College London, London, UK; e MRC Clinical Trials Unit at UCL, University College London, London, UK; f Department of Urology, Ashford and St. Peter's Hospitals NHS Foundation Trust, St. Peter's Hospital, Chertsey, UK; g Department of Urology, Chelsea and Westminster Hospitals NHS Foundation Trust, London, UK; h Department of Urology, The Hillingdon Hospitals NHS Foundation Trust, Uxbridge, UK; i Department of Radiology, The Hillingdon Hospitals NHS Foundation Trust, Uxbridge, UK; j Department of Urology, University Hospital Southampton NHS Foundation Trust, Southampton, UK

**Keywords:** Prostate cancer, Clinical trials, Cohort multiple randomised controlled trial, cmRCT

## Abstract

**Introduction:**

The traditional double blind RCT is the ‘gold standard’ trial design. For a variety of reasons, these designs often fail to accrue enough participants to conclude. This is particularly challenging in localized prostate cancer. The cohort multiple randomised controlled trial (cmRCT) trial design may represent an alternative approach to delivering robust comparative data in prostate cancer.

**Patients and methods:**

IP3-PROSPECT is a cmRCT designed to test multiple prostate cancer interventions from eligible men in one cohort. Key to the design is two points of consent. First, at point of consent one, men referred for prostate cancer investigations are invited to join the cohort. They may then be randomly invited at a later date to consider an intervention at point of consent two. In the pilot phase we will test the acceptability and feasibility of developing the cohort.

**Results:**

Acceptability and feasibility of the study will be measured by a combination of quantitative and qualitative methods. The primary outcome measure is the rate of consent to inclusion to the IP3-PROSPECT cohort. Secondary outcome measures include the completeness of data collection at sites and return rates of patient questionnaires. We will also interview patients and healthcare professionals to explore their thoughts on the implementation, practicality and efficiency of IP3-PROSPECT.

**Conclusion:**

The IP3-PROSPECT study will evaluate the cmRCT design in prostate cancer. Initially we will pilot the design, assessing for acceptability and feasibility. The cmRCT is an innovative design that offers potential for building a modern comparative evidence base for prostate cancer.

## 1 Introduction

Level one evidence is traditionally obtained from randomised controlled trials (RCT). Most commonly one or two new treatment(s) compared to an established treatment or suitable control. Where possible, blinding is maintained so that neither the patient nor clinical team know who is taking what, until the end of the study (‘double-blind’), often supported by use of a placebo. The treatment allocation is based on chance and the blinding minimises bias in how patients or the clinical team behave during their treatment and after. When one of the treatments is surgery this design may not always be ethical or practical. Unfortunately, in trials involving surgery or complex interventions, a variety of factors can lead to the failure to accrue sufficient participants in many traditional RCT designs. This has been particularly highlighted in localized prostate cancer RCTs, where 12 RCTs evaluating different interventions in localized prostate have failed to recruit, many in the last 5 years [1]. The same issue has occurred in trials of localized bladder cancer (surgery versus radiotherapy) [2] and renal cancer (surgery versus surveillance; surgery versus ablation) [3,4] as well as other non-urological disease spaces [[5], [6], [7]].

## 2 Rationale for study

Recently published RCTs have reinforced the limitations and uncertainty of current diagnostic and therapeutic pathways for men with prostate cancer [8]. Three RCTs (PLCO [9], EPSRC [10] and CAP [11]) compared screening (10 yr lead-time bias) to no screening and demonstrated no overall survival benefit, although EPSRC showed a small prostate-cancer specific survival benefit. However, there was significant over-diagnosis and over-treatment [11,12]. Another two RCTs compared surgery versus watchful waiting. SPCG4 showed improved all-cause and prostate-specific mortality in unscreened men with absolute reductions of 6% at 15 years [13]. PIVOT showed no improvement in overall or prostate cancer-specific mortality in men detected in the early PSA-screening era (absolute mortality differences <3%) [14,15]. Equally, preliminary results from a trial of external beam radiotherapy showed no benefit in overall or disease-specific mortality compared to watchful waiting among men with non-PSA detected clinically localized prostate cancer [16]. The recent ProtectT RCT showed no cancer specific survival differences between the three arms of radical prostatectomy, radical radiotherapy and active monitoring in men diagnosed as a result of PSA screening; ProtectT did show improvements in metastases-free survival using radical therapy over active monitoring [17]. These studies demonstrate that there is significant uncertainty around treatment options for men with prostate cancer and therefore a need for further comparative effectiveness research to guide treatment options.

The problem is that doing trials in which the interventions are significantly different to usual practice and in which one arm contains a physical intervention or surgery are, and have always been, difficult. We recently conducted a review of all RCTs in prostate cancer treatment evaluations that have failed to accrue over the last 10 years [1]. We found five United Kingdom (UK) and six international RCTs evaluating novel interventions in non-metastatic prostate cancer that closed prematurely due to slow recruitment usually resulting from lack of patient and physician equipoise. This was despite pre-RCT feasibility questionnaires and surveys demonstrating that there was apparent equipoise on the research question to warrant these RCTs.

There have been certain exemplar cases that have succeeded such as the ProtecT study [18] and PIVOT [19]. However, the cost to achieve this has been high (ProtecT £25 M; PIVOT $8 M). Moreover, they have been slow to initiate (2-4 yrs) and report (10-15 yrs) leading to physicians questioning their external validity when they are published [[20], [21], [22]]. A recent RCT funded by the NIHR-HTA in the UK, called PART, struggled to accrue requiring an extension and then showed a 22% non-compliance rate in the radical prostatectomy arm [23]. Another, SPCG-15, run by the very successful Scandinavian Prostate Cancer Group has also failed to accrue [24]. The SPCG successfully conducted SPCG4 – the RCT of watchful waiting versus surgery in a group of men recruited 20 to 30 years ago - so this points to the current climate for running comparative effectiveness research having changed since SPCG4, PIVOT and ProtecT. The problems with RCTs in surgery, particularly, have been comprehensively reviewed recently in an UK National Cancer Research Institute review [25].

Persisting with our current model of acquiring evidence in this space therefore has a high chance of failing. The alternative, because we struggle to recruit to standard RCTs, is to give up and stop further innovations being evaluated within robust RCTs. Neither option is appropriate or acceptable. The third option is to determine whether a novel trial design might work [26]. The European Medicines Agency (2010) states that “*One approach for a given study might be to consider the study population as a cohort in which to implement the most appropriate design for each objective, thus ensuring alignment of each objective to the best possible design and analysis*” [27]. Further, the Medical Research Council (MRC) Guidelines for Evaluating Complex Interventions (2008) states that novel designs could be justified in areas of difficulty in conducting standard RCTs [28]. *Namely, “Preference trials and randomised consent designs*: *Practical or ethical obstacles to randomisation can sometimes be overcome by the use of non-standard designs*. *Where patients have very strong preferences among treatments, basing treatment allocation on patients' preferences, or randomising patients before seeking consent, may be appropriate.*” Certainly, in the area of prostate diseases, high quality clinical research and investigation of novel strategies in patients with prostate diseases has been sufficiently difficult in the recent past, so that the evaluation of a new way of running clinical trials is warranted.

### 2.1 The Imperial Prostate 3 (IP3)-PROSPECT Study

Our study will explore a trial design called by originators Relton and Nicholl the cohort-multiple RCT (cmRCT) [29]. This design has been used in a number of disease areas, both benign and cancer. In total, a recent systematic review showed that there were 18 ongoing cmRCT studies with 6 in the UK [30]. We have chosen prostate conditions since they are extremely common and, if malignancy occurs, the majority of men with the disease are regarded as living with a chronic condition due to its long natural history and in which innovative approaches, interventions, treatments or changes in management might have a significant patient benefit and impact on the NHS. It therefore fits the cmRCT design very well. Nonetheless, the lessons we learn in this study will be of relevance to other disease spaces.

We want to test the acceptability and feasibility of the cmRCT in the prostate pathway. As this is the first time that we are trying out this method we need to first pilot it. In the first part of the study, we want to evaluate the following: What is the accrual rate? What do patients and their healthcare professionals think of the cmRCT design? Is the data we collect robust? What are the resource requirements of such a study? We will then test a number of novel interventions or changes in the pathway and compared them to standard care in the cohort that we recruit.

Patient and public involvement activities included presenting the issues around this to 40 members of the Mount Vernon Prostate Support Group and with the Maggie's Cancer Centre Prostate Support Group (Charing Cross Hospital). All the men present stated a reluctance to participate in head-to-head RCTs, but discussions around the novel cmRCT design were positive. A number of men in this focus group commented that the cmRCT would lead to greater patient access to trials and innovative therapies. The results of these deliberations were published [31].

Our group also conducted a consensus meeting of 63 members to consider trial designs for localized prostate cancer. There were five patient representatives alongside triallists and methodologists as well as physicians [1]. The patient and public representatives provided key input into all discussions at whole group level and within break-out sessions. They again stated a reluctance to participate in head-to-head RCTs, whilst accepting the need for robust data to change practice. The group reinforced the need to consider alternative trial designs that might be more acceptable to patients and support recruitment [1]. This proposal has thus originated from patient concerns and has a design largely driven by involved patients and public as well as key trial methodologists and experts in the field.

## 3 IP3-PROSPECT Aims and Objectives

In the pilot phase of IP3-PROSPECT we are interested in investigating:

a) the acceptability and feasibility of establishing the cohort of men, i.e. putting the questions of point of consent One to men being referred for investigation of prostate cancer, and.
b) observing the cohort over the study period and collecting data on the participants pertaining to their disease, their treatment and their health status.

## 4 Trial outcomes

We will demonstrate these aims and objective – shown in more detail in Table 1, Table 2 – through a combination of quantitative and qualitative methods outcomes.

**Table 1:** Aims, objectives and outcomes for the acceptability of the cmRCT design in localized prostate cancer.

Acceptability		
Aims	Objectives	Outcomes
To determine what proportion of men with a clinical suspicion for prostate cancer will participate in a cmRCT.	To evaluate proportion of patients approached who agree to participate in the longitudinal cohort. This will be done by calculating the participation rates from men approached for invitation to IP3-PROSPECT.	Rate of consent to inclusion to IP3-PROSPECT cohort at the original point of contact by the research team. This will be calculated on an ongoing basis and will be reviewed at 6 months and one year from opening and at the end of the study period.
To explore barriers and facilitators to implementation of a cmRCT in order to improve and inform patient and/or physician trial information, study processes, interventions, and recruitment and retention of patients. This will be carried out by qualitative assessments in the following areas.	To investigate by interview the patient experiences and perspectives on; - Trial participation, - The point at which men are approached by the research team to enter the cohort, - Barriers and facilitators to consent to participate in the cohort, - Barriers and facilitators to consent to future selection to undergo a new healthcare intervention, - Acceptability of monitoring of health status and the tools used to do this in the cohort.	To investigate by interview the experiences and perspectives of patients who; - Consented to inclusion in the cohort study, - Declined to enter into the cohort, - Consented to inclusion in the cohort initially, but who subsequently requested to leave the cohort.
	To investigate by interview the experiences and perspectives of healthcare professionals (doctors, nurses and admin staff) on; - Trial design and information to patients and healthcare professionals, - Feasibility of future random invitation of participants to interventions, - Tools used for measuring health status.	To investigate by interview the experiences and perspectives of healthcare professionals in regard to the implementation, practicality and efficiency of IP3-PROSPECT.

**Table 2:** Aims, Objectives and Outcomes for the feasibility of delivering a cmRCT in localized prostate cancer.

Feasibility		
Aims	Objectives	Outcomes
To determine the feasibility of recruitment and logistical implementation of IP3-PROSPECT in different data collecting centres based in different institutions. This will be broken down into the following sub-questions	Evaluating how the patients are successfully identified and the option of how inclusion in the trial is presented to them	Evaluation of the number of men approached to enter IP3-PROSPECT against the number of men referred to the participating centres for investigation of prostate cancer.
	Evaluate patient questionnaire response rates for pre-treatment quality of life.	We will conduct a review of the pathway by which we approach men to invite them to the cohort. Part of this will be included in the qualitative interviews with men and healthcare professionals. Particular points of interest will be the timing of consent process, the trial personnel who gain consent, and the number of men who give consent who are subsequently not diagnosed with prostate cancer.
	Evaluate patient questionnaire response rates at pre-determined intervals following on from the point of recruitment into the trial to determine how we might promote optimal patient response rate and improve data collection.	Participants will be given a standard Quality of Life Questionnaire (EQ5D-5 L) at the point at which they consent to inclusion into IP3-PROSPECT. We will calculate the completeness of this data at 6 and 12 months after opening and on an ongoing basis as long as the study is open.
	To evaluate completeness and fidelity of clinical data on the men who participate in the cohort.	All participants will be asked to complete questionnaires on disease specific quality of life at the following points; - Recruitment to cohort, - 0-6, 6, 12, 18, 24 months from recruitment to cohort, - Yearly questionnaires thereafter during inclusion in the cohort if trial remains open (i.e., post pilot phase). IP3-PROSPECT will calculate the rates of response from participants with these questionnaires.
	To evaluate completeness and fidelity of clinical data on the men who participate in the cohort.	Feasibility of collecting data from the participating centres evidenced by the completeness of data for cohort participants including; i. Subject Data: age, co-morbidities, ECOG/WHO Performance Status, ethnic risk, family risk. ii. Disease Characteristics: PSA, MRI (volume, score), biopsy findings (cancer or not, grade if cancer, length of maximum cancer, other pathology), TNM stage if cancer. iii. Treatment Data: modality, follow-up, adjuvant and salvage treatments, mortality. We will conduct this analysis at one year after opening the IP3-PROSPECT and yearly thereafter in order to monitor any trends in improving or faltering data accrual on participants, as long as the study is open.

### 4.1 Acceptability

To determine the acceptability of the cmRCT design we will calculate the rate of consent to inclusion to the IP3-PROSPECT cohort. This will be calculated on an ongoing basis and will be reviewed at 6 months and one year from opening and at the end of the study period.

We will also investigate by interview the experiences and perspectives of: patients who consented to inclusion; patients who declined to enter into the cohort study; and patients who consented to inclusion in the cohort initially and who subsequently requested to leave the cohort. We will likewise investigate, by interview, the experiences and perspectives of healthcare professionals in regard to the implementation, practicality and efficiency of IP3-PROSPECT.

### 4.2 Feasibility

We will evaluate the number of men approached to enter IP3-PROSPECT against the number of men referred to the participating centres for investigation of prostate cancer.

We will also conduct a review of the pathway by which we approach men to invite them to the cohort. Part of this will be included in the qualitative interviews with men and healthcare professionals. Particular points of interest will be the timing of consent process, the trial personnel who gain consent, and the number of men who give consent who are subsequently not diagnosed with prostate cancer.

Participants will be given a standard Quality of Life Questionnaire (EQ-5D-5L) at the point at which they consent to inclusion into IP3-PROSPECT. We will calculate the completeness of this data at 6 and 12 months after opening and on an ongoing basis as long as the study is open.

All participants will be asked to complete questionnaires on disease specific quality of life at specific time points in the pilot study. We will use three self-reporting quality of life validated questionnaires. The responses from the questionnaires will be of value as they will provide an informative vignette of the experience of men after the diagnosis of prostate cancer and we can compare these against the experience of men who are investigated for but not diagnosed with prostate cancer. In terms of evaluating the feasibility of the cmRCT design, IP3-PROSPECT will calculate the rates of response from participants with these questionnaires to assess for feasibility. Questionnaire response rates will also inform our understanding of the acceptability of the cmRCT design study to patients.

We will also assess study feasibility by measuring the completeness of data for cohort participants including; subject data, disease characteristics and treatment data. We will conduct this analysis at one year after opening the IP3-PROSPECT study, and yearly thereafter in order to monitor any trends in improving or faltering data accrual on participants. We will do this for as long as the study is open.

## 5 Trial design

### 5.1 Design

The key features of a cmRCT are:

1. Explicitly consented recruitment of a large cohort of patients with the condition of interest.
2. Regular measurement of relevant outcome measures for the whole cohort prospectively in the long-term.
3. Facility to re-approach cohort participants, who are randomly selected from eligible patients within the cohort, inviting them to undergo intervention of interest to researchers with eligible patients not randomly selected entering the control standard care group (see Fig. 1).

**Fig. 1:**
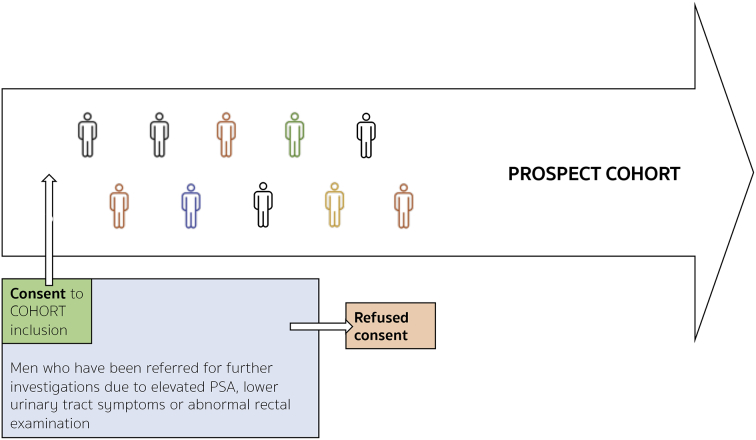

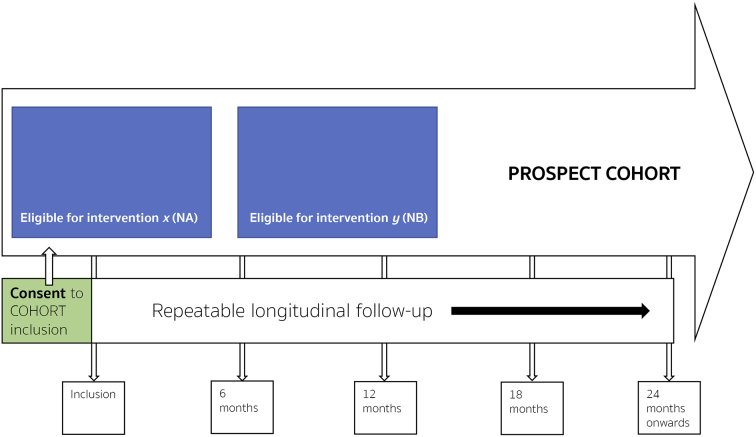

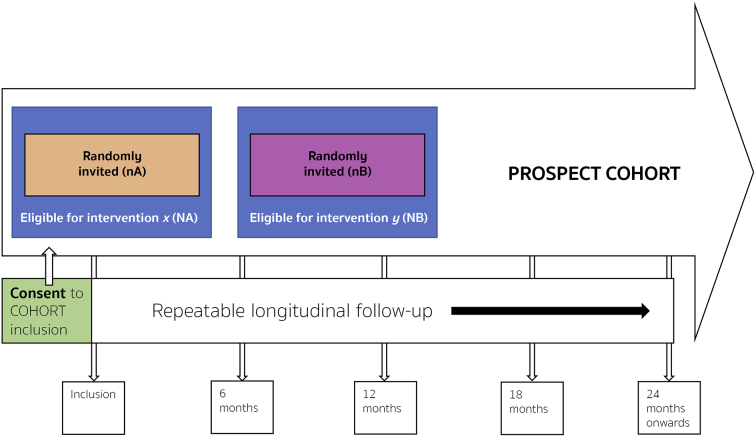

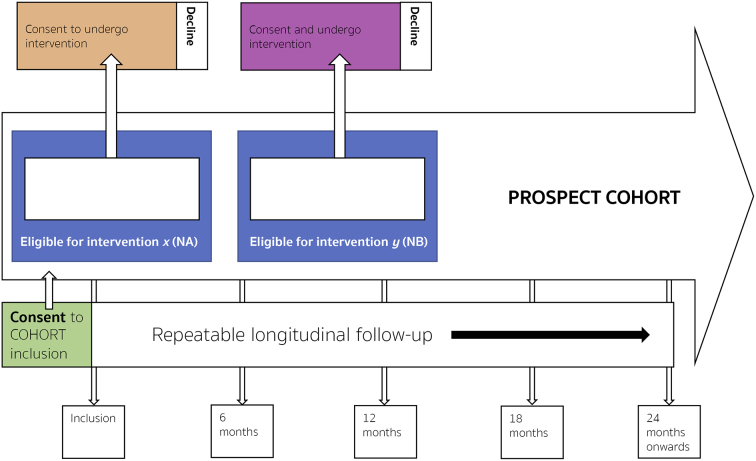
IP3-PROSPECT study flowchart. a) Inclusion of participants to the large heterogeneous cohort at point of consent 1 from men referred for prostate cancer investigations. b) Cohort followed up regularly over time; within cohort there are participants eligible for simultaneous interventions (‘NA’ & ‘NB’). c) Randomised invitation to consider each invitation. Men not randomly invited form the control group. Consent process mirroring normal standard of care. d) Some participants will decline, and some decide to undergo the intervention. Both are kept in the intervention group for analytical purposes. The clinical endpoints are determined by the intervention being tested. Long-term longitudinal follow-up is by national electronic health records. Analysis is by an unmodified intention-to-treat approach. Groups ‘nA’ and ‘nB’ (interventions), would be compared to groups ‘NA-nA’ and NB-nB’ respectively. (Adapted from Relton et al. [29]).
4. “Patient-centred” informed consent. The consent process aims to replicate that used in the routine health care setting. Once the patient has been randomly selected for a randomised novel intervention from the eligible patients within the cohort, the second consent process should include detailed and specific information pertaining to the particular intervention or change in management they are being invited to undergo for comparison. Such information will be written and advised using patient representatives and undergo review following submission to REC.
5. Comparison of the outcomes in the randomly selected patients with the outcomes in eligible patients not randomly selected.
6. Capacity for multiple randomised controlled trials over time within the cohort simultaneously.

This structure can be seen in the cmRCT flow diagram (Fig. 1).

### 5.2 Consent

For men who are participating in the cmRCT there are two points of consent:

#### 5.2.1 Point of consent one

At point of consent one, participants who are referred for investigation of prostate cancer will be asked two questions. The first question relates to whether they are willing to join the cohort and have data collected directly from them over time on a regular basis. This data will include health-related quality-of-life data (at recruitment, 0–6, 6, 12, 18 & 24 months post recruitment), linkage to their medical records so that researchers can know what happens to them over time, and access to other data about them held on national health registry databases. Also, at point of consent one, prospective participants will be asked (second question) whether they agree to being randomly selected in the future to interventions or changes in management in order to compare to standard care. We will explain that this second invitation will be on a random basis. In other words, everyone eligible within the cohort will have the same chance of being randomly selected. The patient would still have the option of saying ‘no’ after the random selection when they are approached.

#### 5.2.2 Point of consent two

The second point of consent is the invitation to undergo an intervention or change in management that the research team wishes to compare to standard care. Participants will have already agreed to the possibility of being invited to undergo intervention at point of consent one (i.e. enrolment into the cmRCT). The participant will have been randomly selected from among all the eligible participants for the given intervention from within the large cmRCT cohort prior to being approached by the trial team. Then, the trial team will approach the participant and invite him to undergo the intervention. This will entail a comprehensive consent process that pertains directly to the intervention being proposed in a patient-centred manner. The participant can agree or refuse to undergo the intervention. If he does not wish to undergo the treatment he will instead undergo the National Health Service (NHS) standard of care treatment and continue under follow-up in IP3-PROSPECT.

Participants undergoing intervention will continue to have follow-up in the same manner as participants who have not been randomised from within the cohort and thus provide outcome data to form the control arm. Comparison of the outcomes of participants who underwent trial intervention against those who did not will allow us to analyse the effectiveness of the intervention in as robust a manner as possible given that the key feature of randomisation when creating the control vs. the intervention arms has been preserved. As such, the control arm of the cmRCT will be similar to the intervention arm in all features, known and unknown, except the intervention of interest. This will allow us to maintain the epistemological superiority of the data we produce for evaluating new tests or treatments whilst getting to this point in a way that might be more acceptable to patients and therefore more likely to be successful and efficient for researchers.

The second stage of IP3-PROSPECT will be to investigate and evaluate in a similarly careful manner the feasibility and acceptability of randomising participants from our cohort of eligible participants to interventions or changes in management that require evaluation, following submission to REC. As part of the cmRCT design, these participants randomly selected are re-approached and invited to consider undergoing the intervention of interest. Patients who are randomly allocated to the control arm will also receive standard of care, and are not informed about their participation in the control arm. This additional consent will be obtained at the time of consent for the cohort study.

## 6 Eligibility criteria

We wish to include all patients referred for investigation of prostate cancer. The inclusion criteria joining the cohort at point of consent one are deliberately broad. Inclusion and exclusion criteria for randomisation to an intervention will differ depending on what that intervention is.

### 6.1 Inclusion criteria

1. Those aged 18 years old and over who are referred for investigations for urinary symptoms or elevated serum prostate specific antigen (PSA) levels or other risk factors for possible prostate malignancy.
2. Those aged 18 years or older with a diagnosis of prostate cancer on active surveillance or referred from another centre for consideration of surgery, radiotherapy or ablative therapy to the prostate
3. An understanding of the English language sufficient to understand written and verbal information about the trial and consent process.
4. Estimated life expectancy of 5 years or more.
5. Signed informed consent.

### 6.2 Exclusion criteria

1. Those who are unable to give informed consent.

## 7 Trial entry

All who meet the eligibility criteria for inclusion in the cohort will be contacted by the dedicated Research Team Recruitment Officer in each participating centre.

Potential participants will be patients who are referred to the respective centre for investigation of their prostate for possible malignancy as part of the NHS suspected cancer referral system. The NHS clinical team will make contact with each man as normal, to organise their first appointment and inform them that the study recruitment may approach them to discuss the trial.

The dedicated Research Team Recruitment Officer will contact the eligible men by telephone before the patient is seen in clinic. The patient will be asked for permission to send him the literature pertaining to the IP3-PROSPECT study including REC approved PIS, ICF and contact information for the research team along with the patient invitation letter. All potential participants will have the opportunity to discuss all aspects of the study with their GP, family members, and their Recruitment Officer prior to the clinic appointment at their participating centre.

Each participant will then be invited to attend a screening and enrollment visit. Here, eligibility will be confirmed and informed consent obtained. Baseline PROMS questionnaires will be collected at this initial visit, including the Expanded Prostate Cancer Index Composite 26 [32], International Prostate Symptoms Score [33], International Index of Erectile Function 15 [34] & EQ-5D-5L [35].

### 7.1 Follow-up visits

Follow-up visits will occur in line with standard of care at 0–6, 6, 12, 18 and 24 months after enrollment. The design and number of visits mirror the standard of care pathway for patients undergoing either focal therapy or radical therapy, so that additional patient burden is minimal.

Following enrolment, all patients will undergo a standard follow-up regimen in the cohort. If they undergo an MRI and biopsy, these usually occur within 6 months of enrolment under standard care. The results of these tests will be collated directly from patient records. If the patient has a cancer diagnosis then the biopsy laterality, Gleason grade and clinical and radiological stage details will be collated in the CRFs. Follow-up information (PSA blood tests, treatments given) will be collated from each follow-up visit directly from the patient records into CRFs. Specific treatment types (active surveillance, surgery, radiotherapy, minimally invasive therapies) or further diagnostic tests (MRI, biopsy) will be recorded into CRFs. Performance status will be collected. Further, patients will be contacted, or sent questionnaires to enquire about any prostate tests or treatments they might have undergone as well as patient reported outcome measures.

### 7.2 Study visit tests

Blood samples for PSA can be collected during a scheduled hospital visit or at a hospital local to the patient or at a community/primary care facility, whichever is more convenient for the patient provided the result is available to the research team. PROMS questionnaires for each visit, can be completed or returned during a scheduled hospital visit or via post/electronic communication. Follow-up imaging will not be protocol led, with appropriate imaging chosen as per the local hospital resources and policies (may include any of prostate MRI, nuclear medicine bone scan, PET-CT/MRI, Whole body MRI or CT chest/abdomen/pelvis). Some patients will undergo further biopsies due to ongoing suspicion of cancer or following treatment as part of their standard care. Some patients will also undergo secondary non-cancer related procedures to manage adverse events from the therapy such as, but not limited to, transurethral resection of prostate, bladder neck incision, urethral dilatation, optical urethrotomy, male-sling or other continence procedures as well as penile implant surgery for impotence. Endoscopic tests or interventions of the lower bowel may also be required. These rates of interventions will be collected.

### 7.3 Trial treatments

Examples of trial treatments offered to eligible participants within the IP3-PROSPECT cohort include, prostate cancer risk calculators, variations in robotic-assisted radical prostatectomy technique, focal therapy techniques and lifestyle modifications prior to, or during prostate cancer treatment. This is not an exhaustive list. It is not possible to provide information on all of the possible interventions that might be evaluated that a man may be invited to participate in, because we do not yet know what they all are. Further treatments may become available in the future which we are not currently able to predict and inform the patient about. Any future proposed intervention will be subject to a full application to the REC to include recruitment into the IP3-PROSPECT study as an eligibility criteria.

### 7.4 Long-term follow-up

At the screening visit, patients will also be asked to give consent for identifiable data to be linked with the national databases (Office for National Statistics (ONS) and Hospital Episode Statistics (HES) database). We will ask patients if they are happy to give consent for their health status to be followed up over time. This will be done by linking the patient's name and NHS number with records held by the NHS and maintained by the NHS Digital, or any applicable NHS information system. This will allow us to track what happens after the study finishes and observe if anyone gets cancer in future and about the type of cancer and the treatment they have had.

We will also ask patients whether or not they give permission to be contacted by a member of the central / local study research team within 10 years of signing their consent form, after the study has ended to assess their willingness to complete a questionnaire about their health status (including details of any other tests and treatment they have had since the study) and quality of life. If the patient decides to take part a member of the study research team may send this request to the patient's home address. This is only done once it is confirmed that the patient is alive, so as not to cause unnecessary distress to relatives of patients who have deceased.

As prostate cancer is often a slow-growing disease which may not progress for many years we will also ask patients if they are happy to keep personal data be stored or accessed for an additional 10 years on the National Health Service Care Register (NHSCR). This is an optional part of consent.

## 8 Statistical analysis

### 8.1 Sample size calculation

We will measure the precision around a conservative 30% anticipated acceptance to participate in the study. 80 patients must agree to join the cohort to show that 30% (95% CI of ±10%) of the eligible patients will accept initial consent with a 95% CI of ±10%, assuming 5% loss to follow up.

## 9 Integrated qualitative outcome measures

### 9.1 Trial participants

The integrated qualitative component of the IP3-PROSPECT trial will explore patients who;

- Consented to inclusion in the cohort study,
- Declined to enter into the cohort,
- Consented to inclusion in the cohort initially but who subsequently requested to leave the cohort,

We will perform structured thematic interviews. Initially, we will aim to interview at least five men who consent to participate in IP3-PROSPECT and at least 5 men who decline to participate in IP3-PROSPECT. We will also ask to interview any men who initially agree to participate in IP3-PROSPECT who subsequently ask to be withdrawn. Recruitment for qualitative interviews will continue until no further themes emerge.

Interviews will be conducted by the researchers who will follow a semi structured Interview Questionnaire Template whilst allowing for some flexibility in the direction and emphasis of the discussion. The interviews will be recorded and transcribed before analysis and theme-based extraction of the reasons behind men's decision regarding inclusion in the cohort. If during the course of the five interviews feedback demonstrates new emerging themes, then consideration will be given to interviewing more men in order to give a broad and fully representative picture of the reasons behind men's decisions.

### 9.2 Healthcare professionals

The opinions of healthcare professionals who regularly look after men with prostate cancer will be sought. We will interview at least 5 doctors and nurses and up to 10 if necessary and up to 5 research and managerial staff. We will conduct interviews at 6 and 12 months from the opening of IP3-PROSPECT. We will perform semi-structured interviews that focus on ethics, implementation, practicality and efficiency of IP3-PROSPECT. There will be a different Interview Questionnaire Template for interviews with healthcare professionals.

## 10 Data management

Data collection will be performed by site personnel, using electronic data capture using the Imperial College London RedCap database. All source data will be recorded in the Clinical Report Form (CRF) and the completed collection forms signed by the Chief / Principal Investigator (CI / PI) or assigned designee and locked. Any changes made to the before signoff are subject to an electronic audit trail. After signoff no changes can be made to the data unless unlocked by the CI / PI.

The study will be monitored periodically by trial monitors to assess the progress of the study, verify adherence to the protocol, International Council for Harmonisation of Technical Requirements for Registration of Pharmaceuticals for Human Use (ICH) E6 Good Clinical Practice (GCP) guidelines and other national/international requirements and to review the completeness, accuracy and consistency of the data.

## 11 Ethical considerations

IP3-PROSPECT will be the first time that the cmRCT design has been piloted for the evaluation of interventions in the prostate pathway. Although the trial design is no longer controversial given its increasingly widespread adoption by clinical researchers worldwide in benign and cancer disease spaces as well as complex interventions, the innovative features of the cmRCT design warrants further consideration of its specific ethical matters, for which expert advice was sought from two ethicists.

### 11.1 Patient information about future interventions at point of Consent 1

The Patient Information Sheet and the Consent Form should not need to include details on the proposed interventions to which the patient may be randomly selected in the future. This is inline with similar ethics committee approved studies and was decided for the following reasons.

• The exact disease status and risk of a disease once diagnosed are not known at the first point of consent for a man.
• It is not possible to provide information on all of the possible interventions that might be evaluated that a man may be invited to participate in because we do not yet know what they all are. Further treatments may become available in the future which we are not currently able to predict and inform the patient about.
• To give prospective participants information about all the possible novel interventions or changes in management for evaluation as embedded randomised interventions to which they may or may not be randomly selected may represent excessive information for the patient.
• Point of Consent 1 gains consent only to collect patient information prospectively and the permission to re-contact in the future if they are eligible for an intervention. IP3-PROSPECT participants categorically cannot undergo any embedded novel interventions or changes in management strategies for evaluation without being approached again by the researchers at Point of Consent 2.
• The principle of consent-for-consent in which patients are asked to consent to a future approach to participate in research (which in itself is subject to further patient information and informed consent) is now being used in a number of disease areas and networks (e.g., BioResource funded by the NIHR).

### 11.2 Participants Consented for possibility of future intervention who are not approached for embedded randomised studies

All IP3-PROSPECT participants will have been consented at Point of Consent 1 for the possibility of being selected in the future for an intervention. Many of these men will not be invited to participate in embedded randomised studies.

• Through the consent process and the PIS we will make clear the structure of the IP3-PROSPECT cmRCT, such that participants are aware that they might not be approached for an embedded intervention as they may be ineligible for interventions being trialed, or they may be eligible but not approached due to the randomised process.
• Through the consent process and the PIS we will make clear that the treatments and interventions being trialed are not of proven benefit to patients. This is why we, as researchers, are seeking information on the embedded interventions. Interventions trialed within the cmRCT format should only be interventions around which there is clinical equipoise. Therefore, it must be clear to patients that the usual care management and treatment they receive will under no circumstances be sub-optimal or compromised as a result of their participation in IP3-PROSPECT within the longitudinal cohort element of the study.
• Through the consent process and the PIS we will make clear that patients who are not selected to embedded randomised interventions in IP3-PROSPECT will continue to experience the standard of care management according to their treating doctors and multi-disciplinary medical team.
• Patients in our focus groups emphasised the fact that participation in the cohort was something that they might derive benefit from. For instance, access to researchers and additional patient reported outcome measures reporting, and that such participation might help others through outcomes measures that might not otherwise be collected and reported.

## 12 Discussion

The cohort-multiple RCT (cmRCT) or the Relton/Nicholl design [29] is also known as the Trials WithIn Cohorts (TWiCS). Others have recently supported the evaluation of such designs and many RCTs are now successfully underway using it [30].

This approach is currently being evaluated in ethics committee approved studies in a variety of diseases, such as depression (NIHR-HTA CASPER, DEPSY) [36,37]; (NIHR DiRECT (Exeter) [38]), obesity [39,40], falls in the elderly (NIHR-HTA REFORM) [41], cognitive behavioural therapy in schizophrenia (Canadian Institute of Health Research [42]), prevention of cardiovascular disease through a polypill [43,44], ulcerative colitis (NIHR-HTA CONSTRUCT) [45], elderly care (NIHR HS&DR 12/130, CLASSIC (Salford) and scleroderma [46].

The ‘Roberts Study’ in bladder cancer and the MRC (UK) funded NEST Study in kidney cancer are both cmRCTs in Kings College London (REC: London - Fulham Research Ethics Committee (REC) 17/LO/1975 [47] and The Royal Free Hospital (East Midlands - Derby REC 19/EM/0004), respectively [48].

In the Netherlands the cmRCT design has also been approved for the investigation of new cancer interventions. The University of Utrecht is successfully running cmRCTs in colorectal cancer (alongside the Dutch Colorectal Cancer Group), breast cancer and cancers with spinal metastases (PICNIC) [[49], [50], [51], [52], [53], [54], [55]]. The Utrecht group have successfully recruited to all three cohorts with 80–90% of those patients approached so far accepting both consent to the cohort and consent to future random selection for new (but un-named) treatments. So far, between October 2013 and July 2016, they have recruited 1308 participants. In this period, 1308/1486 (88%) patients who were invited for participation in UMBRELLA consented to cohort participation. Of these patients, 1138 (87%) gave broad consent for randomisation to future interventions. Return rate for PROMs at baseline was 80% and varied from 67 to 74% during follow-up [49,56].

The cmRCT design and longitudinal framework seems to be successful in conditions where patients affected may transition from different health states over time. The cmRCT design also works well in spaces where Patient Reported Outcome Measures (PROMs), healthcare utilisation and vital status are important outcome measures. Research of patient outcomes in non-metastatic prostate cancer and benign prostate disease is similar to the outcomes of interest in chronic diseases in many respects and as such we believe the cmRCT will lend itself well to the investigation of novel interventions in prostate conditions. If successful, the design could be evaluated in other disease processes in which RCTs are traditionally difficult to deliver to.

Our cmRCT approach has several other potential advantages in that it might allow greater access to RCTs than the current trial system since all eligible participants have an equal chance of being recruited. It might also avoid disrupting existing service provision as well as being able to run trials in a cost-efficient manner [57]. The cmRCT design that we propose represents a potential paradigm shift in the way we might conduct comparative effectiveness research and will allow us to investigate new interventions using a robust method in difficult-to-recruit disease spaces.

Our proposal seeks to reverse the order in which the question and the framework are realised. Typically, “infrastructure” follows a “question” and the infrastructure is disbanded after the trial. We propose to first establish the infrastructure (cohort) in a defined geographic population with case ascertainment, PROMs for functional and vital status, linkage to local institution records for prostate disease status, linkage to national databases for healthcare utilisation and linkage to death registries. Key questions could in future be addressed by embedding multiple RCTs into this infrastructure such as refining surgical techniques; methods to manage side-effects of different treatments; monitoring strategies after different therapies; surgical methods; and pre-operative targeted molecular therapies.

A Lancet editorial discussed the virtues of trial designs that consider multiple interventions, at the same time showing that trial costs could be halved by doing so [58]. So for instance, if we were just to run 3 separate RCTs then each trial might cost an estimated £1–2 million or more if a commercially sponsored drug trial. Our trial design, if successful, would allow additional research questions to be answered at less cost to funders and in a more timely fashion to benefit patients and the NHS. A further attraction of the cmRCT that warrants explicit consideration here is the beneficial consequences on power calculations and rate of recruitment to prospective trials from already having the control group in place. This will allow quicker completion of trial accrual, faster publication of results, increased trial efficiency and reduced trial waste and redundancy. These are important, as there has been a lot of debate recently about the delays that are incurred through trial set-up [59,60].

We expect there to be some challenges due to the innovative nature of the study. Similarly to all clinical trials a slow accrual rate may lead to trial failure or if a trial completes, the results being irrelevant by publication. This too is true for a cmRCT design. For example, if the rate of accrual to the cohort is low at point of consent 1, then the cohort may be of insufficient size to trial proposed interventions. Further, the rate of acceptance to undergo interventions at point of consent 2 may be lower than expected. In other words, there may be crossover from the intervention group to the control group, if the intervention is unacceptable to the participant. If using a standard intention-to-treat analysis, it is possible that the statistical power is so negatively affected that the sample size required to test the efficacy of said interventions becomes so large as to make doing so unfeasible. Our pre-trial focus groups [31] found that participant trust and a close relationship with the trial team is vital to encourage participation in such trials. Since trial commencement we strove to build and maintain these relationships.

### 12.1 Study limitations

The IP3-PROSPECT protocol has some limitations. However, these are inherent to trialling a new trial design and demonstrated the need for the pilot phase of the study. First, the cmRCT has not yet been tested in prostate cancer and the design may be unacceptable to this group of patients. However, when used in other disease states patient accrual is good and thus there is cause for optimism here. Second, the design, with its large cohort of patients, may elicit a large enough follow-up burden to make its uptake unfeasible. Again, there are numerous examples of the cmRCT design being used in a multi-centre setting in other disease states and thus this may not come to be. Third, Health Research Authority (HRA) currently requires a separate protocol as well as research and ethical application for each intervention arm. This detracts somewhat from an initial proposed advantage of cmRCT. The design should in theory enable for the rapid setup and closing down of intervention arms. In turn this allows for swifter and more cost effective delivery of trials. This requirement therefore may cause some delays to trial set up however advantages remain in the constancy of trial infrastructure in maintaining the overall study cohort. The requirement also ensures that each arm is ethically robust.

## 13 Conclusions

The IP3-PROSPECT study will evaluate the cmRCT design in prostate cancer. Initially we will pilot the design, assessing for acceptability and feasibility. If successful, we will introduce multiple as of yet unknown randomised interventions. The cmRCT is an innovative design that offers potential for building a modern comparative evidence base for prostate cancer.

### 13.1 Trial status

National ethical “approval” has been granted by Fulham Research Committee (REC reference 20/LO/0459). The study is registered with clinicaltrials.gov (NCT04400656) and ISRCTN (11095538) and started recruiting in September 2020.

## Funding

Wellcome Trust Senior Clinical Research Fellowship. Grant code: 204998/Z/16/Z.

## Author declaration

1) We wish to draw the attention of the Editor to the following facts which may be considered as potential conflicts of interest and to significant financial contributions to this work.

HUA's research is supported by core funding from the 10.13039/100005622United Kingdom's National Institute of Health Research (NIHR) 10.13039/501100013342Imperial Biomedical Research Centre. HUA currently receives funding from the Wellcome Trust, Medical Research Council (UK), Cancer Research UK, Prostate Cancer UK, The Urology Foundation, BMA Foundation, Imperial Health Charity, NIHR Imperial BRC, Sonacare Inc., Trod Medical and Sophiris Biocorp for trials in prostate cancer. HUA was a paid medical consultant for Sophiris Biocorp in the previous 3 years. HUA receives fees for proctoring in cryotherapy (Boston), Rezum (Boston) and HIFU (Sonacare Inc).

1) We confirm that the manuscript has been read and approved by all named authors and that there are no other persons who satisfied the criteria for authorship but are not listed. We further confirm that the order of authors listed in the manuscript has been approved by all of us.
2) We confirm that neither the entire paper nor any of its content has been submitted, published, or accepted by another journal. The paper will not be submitted elsewhere if accepted for publication in the Journal.
3) We confirm that we have given due consideration to the protection of intellectual property associated with this work and that there are no impediments to publication, including the timing of publication, with respect to intellectual property. In so doing we confirm that we have followed the regulations of our institutions concerning intellectual property.
4) We confirm that any aspect of the work covered in this manuscript that has involved either experimental animals or human patients has been conducted with the ethical approval of all relevant bodies and that such approvals are acknowledged within the manuscript.
5) We understand that the Corresponding Author is the sole contact for the Editorial process (including Editorial Manager and direct communications with the office). He/she is responsible for communicating with the other authors about progress, submissions of revisions and final approval of proofs.

## Declaration of Competing Interest

Professor Ahmed's research is supported by core funding from the United Kingdom's National Institute of 10.13039/100005622Health Research (NIHR) 10.13039/501100013342Imperial Biomedical Research Centre. Ahmed currently receives funding from the Wellcome Trust, Medical Research Council (UK), Cancer Research UK, Prostate Cancer UK, The Urology Foundation, BMA Foundation, Imperial Health Charity, NIHR Imperial BRC, Sonacare Inc., Trod Medical and Sophiris Biocorp for trials in prostate cancer. Ahmed was a paid medical consultant for Sophiris Biocorp in the previous 3 years.
